# What is Known Regarding the Participation of Factor Nrf-2 in Liver Regeneration?

**DOI:** 10.3390/cells4020169

**Published:** 2015-05-20

**Authors:** José A. Morales-González, Eduardo Madrigal-Santillán, Ángel Morales-González, Mirandeli Bautista, Evila Gayosso-Islas, Cecilia Sánchez-Moreno

**Affiliations:** 1 Laboratorio Medicina de Conservación, Escuela Superior de Medicina, Instituto Politécnico Nacional, Plan de San Luis y Díaz Mirón, Col. Casco de Santo Tomás, Del. Miguel Hidalgo, México D.F. 11340, Mexico; E-Mail: eomsmx@yahoo.com.mx; 2 Escuela Superior de Cómputo, Instituto Politécnico Nacional, México D.F.11340, Mexico; E-Mail: anmorales@ipn.mx; 3 Instituto de Ciencias de la Salud, UAEH, Abasolo 600 Col. Centro, Pachuca 42000, Hidalgo, Mexico; E-Mails: mirandeli@hotmail.com (M.B.); evila_gayosso_islas@hotmail.com (E.G.-I.); ceciliasanchezmoreno@hotmail.com (C.S.-M.)

**Keywords:** Nrf-2, liver regeneration, ARE

## Abstract

It has been known for years that, after chemical damage or surgical removal of its tissue, the liver initiates a series of changes that, taken together, are known as regeneration, which are focused on the recovery of lost or affected tissue in terms of the anatomical or functional aspect. The Nuclear factor-erythroid 2-related factor (Nrf-2) is a reduction-oxidation reaction (redox)-sensitive transcriptional factor, with the basic leucine Zipper domain (bZIP) motif, encoding the *NFE2L2* gene. The Keap1-Nrf2-ARE pathway is transcendental in the regulation of various cellular processes, such as antioxidant defenses, redox equilibrium, the inflammatory process, the apoptotic processes, intermediate metabolism, detoxification, and cellular proliferation. Some reports have demonstrated the regulator role of Nrf-2 in the cellular cycle of the hepatocyte, as well as in the modulation of the antioxidant response and of apoptotic processes during liver regeneration. It has been reported that there is a delay in liver regeneration after Partial hepatectomy (PH) in the absence of Nrf-2, and similarly as a regulator of hepatic cytoprotection due to diverse chemical or biological agents, and in diseases such as hepatitis, fibrosis, cirrhosis, and liver cancer. This regulator/protector capacity is due to the modulation of the Antioxidant response elements (ARE). It is postulated that oxidative stress (OS) can participate in the initial stages of liver regeneration and that Nrf-2 can probably participate. Studies are lacking on the different initiation stages, maintenance, and the termination of liver regeneration alone or with ethanol.

## 1. Liver

The liver is the largest organ of the body, with an approximate weight of 1.5 kg. Homeostatic functions of the body, such as detoxification of waste substances, biotransformation of drugs (acetaminophen), metabolism of carbohydrates, lipids, and proteins, and ethanol metabolism all depend on the liver [1]. The liver is also involved in the biochemical processes of growth, providing nutrients, supplying energy, and reproducing. In addition, it aids in the metabolism of carbohydrates and fats, in the secretion of bile, and in the storage of vitamins [2,3].

## 2. Liver Regeneration

It has been known for years that, after chemical damage to or surgical removal of its tissue, the liver undergoes a series of changes taken together, known as regeneration, which are directed toward the recovery of lost or affected tissue in the functional and anatomical aspect [4]. The exact mechanisms by which the liver regenerates itself are not yet precisely known and have received much attention in recent times, because hepatic regeneration is an excellent experimental model for studying the processes that determine cell proliferation [5].

The mechanisms regulating hepatocyte proliferation have been studied in models of fetal liver, cancer of the liver and liver regeneration. In all of these cases, cellular proliferation comprises a common factor in all of these and it is this phenomenon that is subject to precise regulation on the part of the cell, the tissue, and the organ in general [6].

Among the models most utilized for the study of liver regeneration, the most common is Partial hepatectomy (PH, surgical removal of 70% of hepatic tissue) in laboratory animals (rats, rabbits, guinea pigs, and dogs). Once the liver is divided into lobules, it is possible to remove some of these, which represent the equivalent of 70% of the hepatic tissue, and to leave a remnant of 30%. The growth process of the liver implies the proliferation of the cells of the remnant lobules. The latter does not mean the restoration of the divided lobules: that is, one must not confuse the phenomenon of growth with the restitution of an amputated part (such as amputation of the tail of a lizard) with the phenomenon of liver regeneration. In the latter case, the remnant lobules enter into cellular proliferation until restitution of the functional hepatic tissue that it originally possessed. This growth and proliferation ends at 10–14 h, in all of the species studied, after the surgical procedure [4,6]. One important question not yet answered is to explain the optimal functioning of the remnant liver that maintains the tissue functional. Responding to this question requires a detailed explanation of the mechanisms that initiate, maintain, and terminate liver regeneration. Responding to this type of question will aid in identifying the phases of the regenerative process and will attempt to identify the events that regulate each of these.

Liver proliferation begins 12–14 h after PH, which allows a separation between a pre-replicative (0–14 h) and a replicative state (14–24 h). For the sake of convenience, an initial phase is distinguished, which is the competency phase (or initiation phase), which corresponds to 4 h after the PH (the step from phase G0 to phase G1), and a second progression phase that indicates phase G1 to phase S. Phase G1 begins in diverse areas of the remnant liver parenchyma and its duration is variable; thus, the progression phase is less synchronous than that of the initiation but ends when the cell synthesizes DNA, which possesses peak synthesis in rat at 22–24 h. The succession between the initiation and the progression phase depends directly or indirectly on gene activation/inhibition, activation and regulation of auto- and paracrine circuits, and activation of the machinery required for DNA replication. It is precisely during these early phases of regeneration that the process is more susceptible to being interrupted or altered, because the initial signals can be blocked, giving rise to liver non-regeneration [7,8,9].

In contrast to other organs or tissues, liver regeneration does not depend on a small group of cells. Liver regeneration after PH is carried out by the proliferation of all of the mature cellular populations that comprise the intact organ [10]. This includes the hepatocytes (the main functional cells), biliary epithelial cells (these form the bile ducts), endothelial cells (cells that provide maximal contact between the blood and the hepatocytes), the Kupffer cells (macrophages in the hepatic sinusoids), and the Ito cells (the sole hepatic stellate cells that are localized, which are found under the sinusoids; they synthesize connective tissue, secrete various growth factors, and store vitamin A) [11,12]. All of these cells divide during liver proliferation; the hepatocytes are the first to do this. The kinetics of cellular proliferation differs slightly from one species to another. The first DNA synthesis peak occurs at 24 h, with a second, small peak between 36 and 48 h, until the completion of the entire proliferative process from day 7 to day 10 [4,12].

Studies conducted in animals (dogs and primates) and in humans have established that liver regeneration corresponds in proportion to the amount of liver removed. In small resections (<10%), it follows a proportional response for recovery of the liver. Likewise, it has been observed that when the liver of large dogs is transplanted to small dogs, the liver gradually diminishes in size, at the end being proportional to the size of the new host. On the other hand, when the liver is transplanted to a small from a large dog, the organ grows rapidly (in weeks), again proportional to the size of the new host. This type of study has demonstrated that the hepatic mass is highly regulated and that there are signals that control liver positively, as well as negatively, in order for the latter to acquire a correct size [13]. Similarly, there are diverse mechanisms that are activated during liver regeneration after PH, such as intracellular signaling cascades, which regulate the kinetics of liver regeneration through mitogenic, co-mitogenic, and inhibitory factors, such as the Hepatocyte growth factor (HGF), Transforming growth factor alpha (TGF-α), the Epidermal growth factor (EGF), Transforming growth factor beta (TGF-β), and norepinephrine, among others. Liver regeneration is a complex process that is regulated by diverse intra- and extrahepatic factors, knowledge of their regulation will permit us to apply this knowledge to patients with chronic liver diseases (such as cirrhosis and cancer), where there is dysregulation of liver proliferation, and to afford alternatives to medical or surgical treatment [12].

Oxidative stress (OS) has been proposed as participating in liver regeneration. Aguilar-Delfín *et al.* [14] reported that lipid peroxidation levels in the subcellular fractions of rats with PH or with acute administration of CC1_4_ are qualitatively distinct among subcellular fractions and that this would probably be a normal event in PH-regenerated cells, and that lipid peroxidation could be a modulator of cellular division, exerting an influence on the initiation and cessation of the mitosis involved in liver regeneration. On the other hand, Trejo-Solis *et al.* [15] found a diminution in PH-induced liver regeneration on the administration of vitamin E, the authors concluding that treatment with vitamin E probably could promote an anticipated termination of the preparative events leading to the replicative phase of pH-induced liver regeneration. Ramírez-Farías *et al.* [16] reported that administration of ethanol increases lipid peroxidation levels in rats that had been submitted to PH, giving rise to the inhibition of liver regeneration, while the administration of vitamin E diminished the levels of the lipid peroxidation that produced ethanol, favoring PH-induced liver regeneration.

It is clear that OS plays an important role in liver regeneration and that this depends on the experimental condition that appears that inhibits or favors liver regeneration. Moreover, because Nrf-2 is a modulator of the enzymes that regulate OS, it would surely participate in the proliferative process of the liver.

## 3. Keap1-Nrf2-ARE Pathway

The Nuclear factor-erythroid 2-related factor (Nrf-2) is a redox-sensitive transcriptional factor, with the basic leucine Zipper (bZIP) motif, encoding for the *NFE2L2* gene. In addition, it contains a Cap ‘n’ Collar (CNC) structure. Its activity is regulated by Kelch-like ECH-associated protein 1 (Keap1). In normal concentrations, Nrf-2 is found bound to the Keap1 cytosolic protein, and it is ubiquitinated by the action of Cul 3 for its degradation by proteasomes. On the other hand, on presenting an increase in Reactive oxygen species (ROS), conformational changes are produced in the Keap1 protein, blocking the ubiquitination and proteosomal degradation of Nrf-2. Next, there is a separation between Keap1 and Nrf-2, and the latter translocates to the nucleus, binding to small Maf proteins, and induces its target genes by binding to the antioxidant response element (ARE) [17,18]. The Keap1-Nrf2-ARE pathway is transcendental in the reguIation of various genes of antioxidant and cytoprotector proteins. There are some reports in which their regulation has been demonstrated of the antioxidant enzymes (e.g., catalase, Superoxide dismutase [SOD]), phase-II detoxification enzymes (e.g., Glutathione S-transferase class mu3), Nicotinamide adenine dinucleotide phosphate (NADPH)-generating enzymes (e.g., Glucosa-6-phosphate 1-dehydrogenase) and enzymes for the metabolism of lipids (e.g., Acetyl-CoA oxidase 1) [19,20]. Likewise, evidences suggest that Nrf-2 regulates various cellular processes, such as antioxidant defenses, redox equilibrium, the inflammatory process, apoptotic processes, metabolism, detoxification, and cellular proliferation.

## 4. Nrf-2 and Liver Regeneration

**Figure 1:**
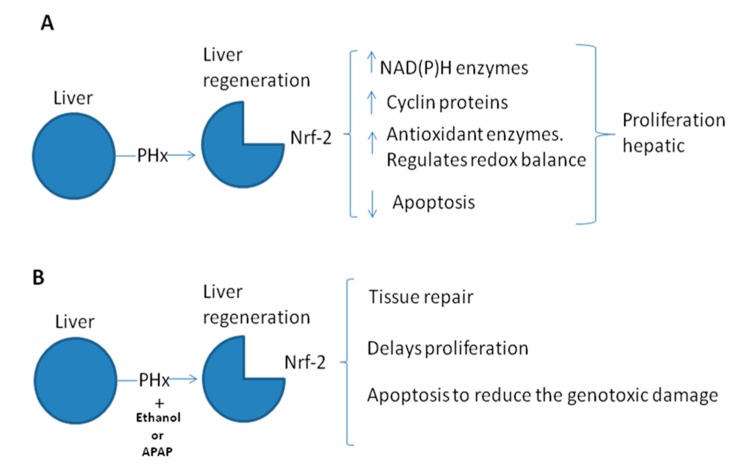
Schematic of the possible participation of nuclear factor-erythroid 2-related factor (Nrf-2) in liver regeneration induced by partial hepatectomy (panel **A**), and when a toxic administered during liver regeneration (panel **B**). PHx: partial hepatectomy; APAP: acetaminophen.

Very few studies have been conducted to investigate the participation of Nrf-2 in the regulation of hepatocyte proliferation during liver regeneration (Figure 1). Zou *et al.* [21] investigated the role of Nrf-2 as the cell-cycle modulator during regeneration, for which Partial hepatectomy (PH) was carried on Nrf-2-null mice, finding that Nrf-2 deficiency did not affect the number of hepatocytes entering the cellular cycle, but rather, gave rise to a delay in hepatocyte mitosis, in addition to the diminution of Cdc2 activity caused by dysregulation of Wee1, Cdc2, and B1 cyclin in its messenger RNA (mRNA) and as well as in the expression of its protein, and also in mRNA levels and in the protein of cyclin A2. These authors concluded that Nrf-2 is required for timely hepatocyte replication by means of adequate regulation of Cyclin A2 and the Wee1/Cdc2/Cyclin B1 pathway during liver regeneration. Similarly, the group of Zou *et al.* [22] demonstrated that Nrf-2 participates in maintaining newly regenerated hepatocytes in a fully differentiated state by ensuring proper regulation of HNF4α, Akt1, and p70S6K during liver regeneration. On the other hand, Hu *et al.* [23] investigated how Keap1 modules the redox cycle and the cellular cycle of the hepatocytes during liver regeneration, for which PH is carried out on Keap1 +/− (Keap1 knockdown) mice, reporting that after PH, Keap1 knockdown caused a delay in entry into the S phase, interruption of S phase progression, and the loss of mitotic rhythm in hepatocyte replication, and that these events are associated with the deregulation of c-Met, EGFR, Akt1, p70S6K, cyclin A2, and cyclin B1 in liver regeneration. With this, the authors demonstrated that Keap1 is a potent regulator of the hepatic redox cycle and the cell cycle of the hepatocytes during liver regeneration.

On the other hand, Fan *et al.* [24], on inducing liver damage with acetaminophen during liver regeneration, reported toxicity during the first 12 h, and later recovery at 24 h, finding a nuclear increase of Nrf-2 after treatment with APAP. Expression of the NAD(P)H enzymes: quinone oxidoreductase 1, the glutamate-cysteine ligase modifier subunit, and hemo oxygenase-1 were induced after 24 h, limiting oxidative damage. In addition, the authors reported an increase at 48 h in cyclin D1 levels, cyclin D-dependent kinase 4, and the proliferating cell nuclear antigen, which are regulators of liver regeneration, which suggests initiation of hepatocytes proliferation and tissue repair. These authors concluded that there is dynamic and coordinated regulation of the Keap1-Nrf2-ARE pathway to regenerate the liver after the damage causes by the APAP. On the other hand, Dayoub *et al.* [25] demonstrated that the Augmenter of liver regeneration (ALR) can be induced by means of the activation of Nrf-2. Therefore, ALR expression by Nrf-2 protects the cells against OS, favoring the proliferative process; this can also exert a beneficial effect of the hepatocyte regarding the damage caused by other agents such as biological or chemical.

There are other studies, such as that reported by Köhler *et al.* [26], which investigated the role of Nrf-2 in liver regeneration and modulation of the genes regulating the cell cycle and apoptosis, they used mice expressing a constitutively active Nrf2 (caNrf2) mutant in hepatocytes. The authors reported in their study that Nrf-2 delays proliferation and induces apoptosis in hepatocytes during liver regeneration. They concluded that these negative effects on Nrf-2 activation in liver regeneration should be considered when Nrf2-activator compounds for the prevention of liver damage.

There are other reports in which the role of Nrf-2 is explored during liver regeneration or in hepatic diseases or its protector effect by diverse agents. Shin *et al.* [27] analyzed the regulator role of Nrf-2 in liver protection by diverse agents (hepatitis B and C viruses, the damage caused by APAP or ethanol), or in diseases such as hepatic alcoholic steatosis, non-alcoholic steatosis, fibrosis, cirrhosis, and hepatocarcinoma. Buitrago-Molina *et al.* [28] reported that liver regeneration and hepatocarcinogenesis was impaired in p21-deficient mice with moderate injury, and that the degree of hepatic lesion and the strength of p21 activation determine its effects on liver regeneration and the development of tumors in the liver, which involves Nrf-2. Wakabayashi *et al.* [29] reported that through transcriptional analyses in Keap1- or Nrf2-disrupted mice, they identified interactions between the Keap1-Nrf2-Antioxidant response elements (ARE) and the Notch1 signaling pathways. The authors found that Nrf2 recognized a functional ARE in the promoter of Notch1; because Notch1 is a cellular proliferation regulator, it would play a role in the modulation of liver regeneration. Beyer *et al.* [30] analyzed the participation of Nrf-2 as a regulator of the cell’s redox homeostasis, this being a cytoprotector factor. The authors reported that liver regeneration was delayed after PH in the absence of Nrf-2, suggesting that the activation of this transcriptional factor could improve liver regeneration in patients with acute or chronic lesion. Madrigal-Santillán *et al.* [31] reported the hepatoprotector effect of *Geranium schiedeanum* in the inhibition caused by ethanol after PH, suggesting that this protection is due both to the trapping effect of the free radicals possessed by *G. schiedeanum* and to the regulation of Nrf-2, concluding that Nrf-2 protects the regenerating liver from ethanol-related damage.

## 5. Conclusions

It is true that there are various reports that speak of the role of Nrf-2 in liver regeneration, regulating proliferative or apoptotic processes, or the activity of antioxidant enzymes, but more must be done, because this is a relatively new field. There is a lack of studies in rat, because those reported are in mouse, and there are differences between the two species. Studies are also lacking on the different stages of liver regeneration (initiation, maintenance, and termination). If the main causal agent of liver damage is ethanol, there is a lack of studies on this toxic agent and the role of Nrf-2 at diverse ethanol concentrations, administration routes, and the diverse stages of liver regeneration. Studies must be conducted utilizing the diverse modulators of Nrf-2, such as the polyphenols, during liver regeneration. All of these studies will aid us in understanding liver regeneration, the role of Nrf-2, as protector the liver from ethanol damage acetaminophen, virus, *etc.*, when it is better for Nrf-2 to activate, or when it is more suitable to inhibit it. There are many questions and few answers.

As Michalopoulos [32] notes in reference to Nrf-2 and liver regeneration: “Unexpected and contradictory signaling functions are often discovered, which in retrospect are better understood as providing a fine balance for optimization of processes which the cell understands better than we do. Nrf-2, as an umbrella protector for the hepatocytes, exercises a very important function in perhaps slowing things down, when needed, to prevent complete catastrophe. This may be one of its most important functions”.
